# Supplementary material for: Facilitators, Barriers, and Educational Preparedness of Early-Career Nursing Graduates Entering Practice in Rural and Remote Areas: A Mixed-Method Study
Source: Nurs Rep. 2025 Nov 20;15(11):410. doi: 10.3390/nursrep15110410 (PMC12655083; doi:10.3390/nursrep15110410)
Supplement: Supplementary file 1 [file nursrep-15-00410-s001.zip › nursrep-3874463-supplementary.pdf]

Qualitative Interview Guide

1. Why did you pursue a nursing career in a rural or remote area?

2. What do you like most about rural and remote nursing?

3. What do you like least about rural and remote nursing?

4. What factors facilitate your ability to function in the setting that you work in?

5. What factors are barriers or impede your ability to function in the setting that you work in?

4. Do you feel like your undergraduate nursing program prepared you to work rurally or remotely? Why or why not?

5.Can you describe your experiences in the undergraduate nursing program that were specific to rural and remote nursing?

6.How do you think your undergraduate nursing program could have better prepared you to work rurally or remotely?

7.What do you think can be done to attract more nurses to work in rural or remote areas?

8.What do you think can be done to retain nurses working in rural or remote areas?

9. What are reasons you would tell other nurses to work rurally or remotely?

10.What tips or advice would you give to nurses considering working rurally or remotely?

Note: This interview may be modified based on the direction of the interview and participants responses.

Modified Nursing Community Apgar Questionnaire (M-NCAQ)

Question	Strong Facilitator (1)	Somewhat of a Facilitator (2)	Neither a Facilitator nor Barrier (3)	Somewhat of a Barrier (4)	Strong Barrier (5)	N/A (6)
1. Nurses who have been born or resided in a rural or remote region	o	o	o	o	o	o
2. Nurses who have been educated in a rural or remote setting (placements, satellite campus, etc)	o	o	o	o	o	o
3. Affordability/Availability of housing	o	o	o	o	o	o
4. Adequate total compensation (relocation allowances, differentials, overtime, benefits, salary, flex scheduling)	o	o	o	o	o	o
5. Access to opportunities for nurses' families (childcare/education, spousal employment)	o	o	o	o	o	o
6. Access to continuing education support (professional development opportunities, scholarships, remote learning)	o	o	o	o	o	o
7. Social and recreational opportunities	o	o	o	o	o	o
8. Nurses' being consulted or involved in decisions regarding healthcare, resources, and equipment	o	o	o	o	o	o
9. Positive workplace culture (recognition, flexible shifts, collegial relationships with other staff/disciplines)	o	o	o	o	o	o

10. Manageable workloads with adequate staffing	○	○	○	○	○	○
11. Robust orientation/welcome program	○	○	○	○	○	○
